# Basiliximab induction alone vs a dual ATG–basiliximab approach in first live-donor non-sensitized kidney transplant recipients with low HLA matching

**DOI:** 10.1093/ckj/sfae236

**Published:** 2024-09-12

**Authors:** Tammy Hod, Shmuel Levinger, Enosh Askenasy, Maya Siman-Tov, Yana Davidov, Ronen Ghinea, Niv Pencovich, Ido Nachmani, Eytan Mor

**Affiliations:** Renal Transplant Center, Sheba Medical Center, Tel Hashomer, Israel; Faculty of Medicine, Tel-Aviv University, Tel Aviv, Israel; Faculty of Medicine, Tel-Aviv University, Tel Aviv, Israel; Renal Transplant Center, Sheba Medical Center, Tel Hashomer, Israel; Faculty of Medicine, Tel-Aviv University, Tel Aviv, Israel; Department of Emergency and Disaster Management, School of Public Health, Faculty of Medicine, Tel-Aviv University, Tel Aviv, Israel; Faculty of Medicine, Tel-Aviv University, Tel Aviv, Israel; Liver Disease Center, Sheba Medical Center, Tel Hashomer, Israel; Renal Transplant Center, Sheba Medical Center, Tel Hashomer, Israel; Faculty of Medicine, Tel-Aviv University, Tel Aviv, Israel; Department of Surgery B, Sheba Medical Center, Tel Hashomer, Israel; Renal Transplant Center, Sheba Medical Center, Tel Hashomer, Israel; Faculty of Medicine, Tel-Aviv University, Tel Aviv, Israel; Department of Surgery B, Sheba Medical Center, Tel Hashomer, Israel; Faculty of Medicine, Tel-Aviv University, Tel Aviv, Israel; Department of Surgery B, Sheba Medical Center, Tel Hashomer, Israel; Renal Transplant Center, Sheba Medical Center, Tel Hashomer, Israel; Faculty of Medicine, Tel-Aviv University, Tel Aviv, Israel; Department of Surgery B, Sheba Medical Center, Tel Hashomer, Israel

**Keywords:** anti-thymocyte globulin (ATG), basiliximab, HLA match, induction treatment, kidney transplantation

## Abstract

**Background:**

Individualizing induction therapy based on immunological risk is crucial for optimizing outcomes in kidney transplantation.

**Methods:**

A retrospective analysis included 157 first live-donor non-sensitized kidney transplant recipients (KTRs). Within this cohort, 96 individuals exhibited low human leukocyte antigen (HLA) matching (5–6 HLA mismatches). The low HLA match subgroup was categorized into 52 KTRs receiving basiliximab alone and 44 recipients treated with a combined single ATG dose of 1.5 mg/kg and basiliximab. The primary endpoint was early acute cellular rejection (ACR) within 6 months post-transplant while secondary outcomes encompassed infection rates, renal allograft function, length of stay (LOS) and readmissions post-transplant.

**Results:**

The incidence of early ACR was decreased for low HLA match KTRs, who received ATG–basiliximab, when compared with low HLA-matched KTRs who received basiliximab alone (9.1% vs 23.9%, *P* = .067). Age was a predictor for rejection, and subgroup analysis showed consistent rejection reduction across age groups. No significant differences were observed in admission for transplant LOS or in peri-operative complications, nor in infections rate including BK and cytomegalovirus viremia, allograft function and number of readmissions post-transplant up to 6 months post-transplant.

**Conclusion:**

In non-sensitized first live-donor KTRs with low HLA matching, a dual ATG–basiliximab induction approach significantly reduced early ACR without compromising safety.

KEY LEARNING POINTS

- This study presents a novel approach to individualizing induction therapy in kidney transplantation.

**This study adds:**

- This study highlights the efficacy of a combined induction regimen comprising basiliximab and a single dose of anti-thymocyte globulin (ATG) compared with basiliximab alone in kidney transplant recipients with low human leukocyte antigen (HLA) match.
- The primary outcome, early acute cellular rejection within 6 months post-transplant, was significantly reduced with the dual ATG–basiliximab approach.
- Importantly, the dual induction strategy did not compromise safety, as evidenced by comparable rates of infections, renal allograft function and peri-operative complications.

**Potential impact:**

- This research underscores the potential of tailored induction therapies to optimize outcomes in kidney transplantation.

## INTRODUCTION

Induction immunosuppression in renal transplantation plays a pivotal role in preventing acute rejection episodes and enhancing long-term graft survival. Among the various induction agents available, anti-thymocyte globulin (ATG) and basiliximab have emerged as key players, each with its unique mechanisms of action and clinical efficacy [1].

ATG, a polyclonal antibody derived from rabbit or horse sources, exerts its immunosuppressive effects by targeting T lymphocytes, making it an effective agent in preventing acute rejection. On the other hand, basiliximab, a monoclonal interleukin-2 receptor antagonist, offers a targeted approach by specifically inhibiting the activation of T cells. Both agents have demonstrated efficacy in reducing the incidence of acute rejection, but their comparative advantages and disadvantages remain the subject of investigation [1]. Research has indicated a lower incidence of rejection when employing ATG, recognized as a more robust induction therapy [2–5]. However, it is noteworthy that this approach is associated with an elevated risk of infections in the short term [6] and an increased susceptibility to malignancies in the long term [7, 8].

Tailoring immunosuppressive therapy based on individualized immunological risk is a crucial approach to enhancing the success of organ transplantation and minimizing complications. Each transplant recipient possesses a unique immunological profile that influences the risk of rejection versus infection. This tailored approach involves assessing factors such as the recipient's immunological history, human leukocyte antigen (HLA) sensitization, HLA matching, presence of donor-specific antibodies and overall status of the immune system [9]. Additionally, donor characteristics such as donor type (live versus deceased) [10], age and peri-transplant ischemic injury leading to delayed or slow graft function are considered [11]. The degree of HLA matching influences the risk of both acute and chronic rejection and plays a pivotal role in determining the immunosuppressive protocol for renal transplantation [12, 13].

Due to the prevalent utilization of living unrelated donor (LURD) transplants in Israel [14] , a substantial proportion of non-sensitized recipients have a low level of HLA matching (5–6 HLA mismatch). Our observations revealed a heightened acute rejection rate in this particular population when employing basiliximab induction. In response to this, we have modified our induction protocol, opting for a combination of single ATG dose and basiliximab. The primary objective was to diminish the incidence of acute rejection, and notably, we have significantly decreased the ATG dosage to mitigate the potential adverse effects associated with higher doses of ATG.

In this study, we compare the 6-month outcomes between basiliximab induction alone and a dual ATG–basiliximab approach in this population. Specifically, our focus is on the acute rejection rate, infection rates, including urinary tract infections (UTI), bacteremia, BK and cytomegalovirus (CMV) viremia, as well as renal allograft function. Additionally, we compare the length of stay (LOS) for transplant admissions and the overall number of readmissions post-transplant between the two groups.

## MATERIALS AND METHODS

### Study population and design

Clinical and biochemical data retrieval was conducted with the assistance of MDClone^©^ software, a data extraction and synthesis tool intricately connected to the medical records of patients treated within our institution (http://www.mdclone.com). To ensure data accuracy and reliability, the information collected through MDClone underwent rigorous manual assessment and validation. Supplementary details were obtained from relevant clinical records. The study received approval from the local ethics committee (Institutional Review Board approval number: SMC-7053-20).

The original dataset comprised 264 transplant recipients who underwent renal transplantation at Sheba Medical Center between August 2019 and August 2023. Among them, 234 underwent their first renal transplant, while 30 underwent a second to fourth renal transplant. Subsequently, six recipients with first-year graft loss and five who passed away during the initial post-transplant year were excluded from the analysis. Furthermore, 41 recipients of deceased donor renal transplants, 37 sensitized recipients with antibodies to HLA (with and without donor-specific antibodies), 4 who underwent ABO incompatible renal transplants, 4 recipients who underwent a combined bone marrow–kidney transplant and an additional 10 recipients who underwent their second or third renal transplant were excluded from the study.

**Figure 1:**
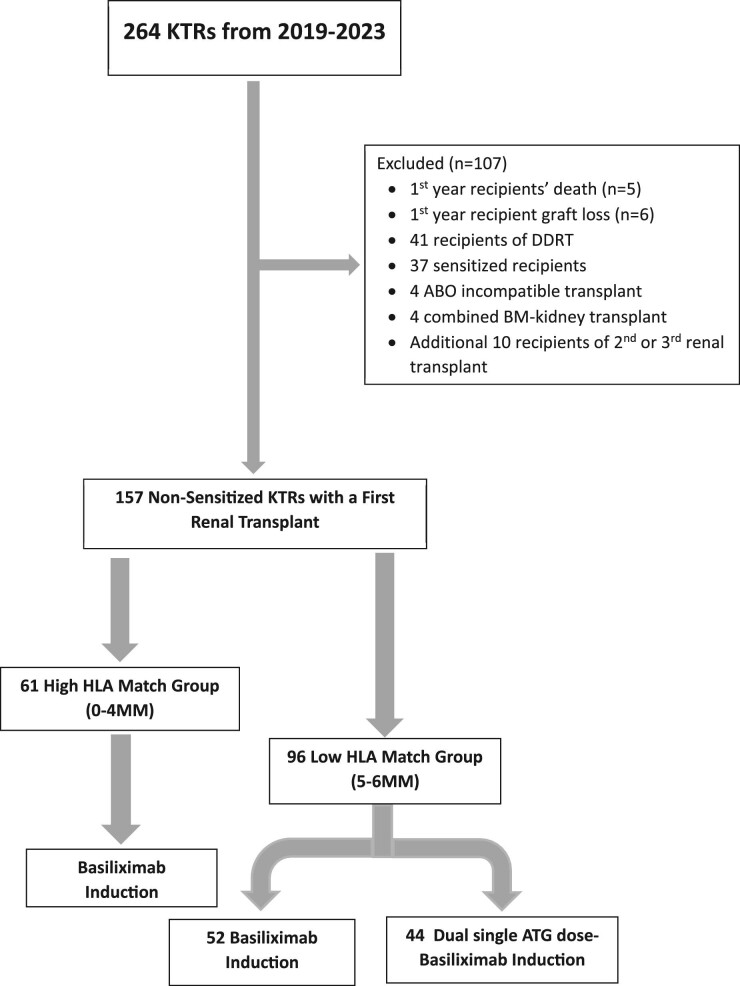
CONSORT diagram.

The final study cohort comprised 157 non-sensitized kidney transplant recipients (KTRs) who underwent their first live-donor renal transplant at Sheba Medical Center. Of these, 61 fell within the 0–4 HLA mismatch range with their donors, constituting the high HLA match group, while 96 had 5–6 HLA mismatches, categorizing them as the low HLA match group. All recipients in the high HLA match group received induction therapy with basiliximab. In the low HLA match group, 52 received basiliximab and 44 were subjected to a combined single ATG dose–basiliximab regimen for induction, as illustrated in Fig. 1.

### Induction therapy

The basiliximab induction protocol involved administering 20 mg intravenously in accordance with the manufacturer's guidelines. This entailed the first infusion prior to graft reperfusion, followed by a second infusion on Day 4. The manufacturer did not recommend any premedication or dose adjustments.

For the combined ATG–basiliximab induction, a single dose of 1.5 mg/kg body weight of rabbit ATG was administered intravenously intraoperatively before graft reperfusion. Subsequently, two additional doses of 20 mg basiliximab were given postoperatively on Day 0 and Day 4, as described above.

### HLA analysis

Blood samples from potential donors and recipients were dispatched to the Tissue Typing Laboratory at Sheba Medical Center for HLA typing prior to transplantation. Utilizing the NGS-go MX6-1 kit, the next-generation sequencing (NGS) method was employed to perform HLA genotyping for six specific loci (A, B, C, DRB1, DQB1 and DPB1). Subsequent to the analysis of sequencing data (FASTQ files) using NGSengine software from GenDx in the Netherlands, results pertaining to HLA-A, HLA-B and HLA-DRB1 alleles were compiled. Comparison of HLA typing data for each allele between donor and recipient pairs facilitated the determination of a 0–6 HLA match/mismatch based on allele-level compatibility across all six loci.

### Maintenance immunosuppression

At our medical center, the standard maintenance immunosuppression regimen for KTRs comprises a calcineurin inhibitor (usually tacrolimus), an anti-metabolite [usually a mycophenolate-based drug, mainly mycophenolic acid (MPA)], and prednisone, as described previously [15]. For KTRs with a low immunological risk of rejection, early steroid withdrawal (ESW) is implemented 5–8 days after transplant, and the maintenance regimen thus consists of tacrolimus and MPA. Conversion to a mammalian target of rapamycin (mTOR) inhibitor (sirolimus or everolimus) is instituted according to the patient's risk of malignancy and lack of tolerance to CNIs.

### Primary and secondary outcomes

The primary endpoint of this study was the rate of early acute cellular rejection (ACR), whether biopsy-proven or clinically proven. Clinically proven ACR was determined by a rapid increase in serum creatinine (Scr) without evidence of a prerenal/nephrotoxic injury, highly suggestive of rejection, which was clinically confirmed by a rapid response to intravenous steroid pulse. Early ACR, for the purposes of this study, was specifically defined as any cellular rejection episode occurring within the initial 6 months post-transplantation. In our study cohort, we identified a total of 20 cases of early ACR: 13 were confirmed by biopsy, while 7 were diagnosed clinically. Additionally, we assessed several secondary outcomes, including the UTI and bacteremia rate during the first month and from 1–6 months post-transplant, and the presence of CMV and BK virus viremia. CMV and BK viremia were identified through serum polymerase chain reaction, with a threshold of above 1000 copies/mL for CMV and above 500 copies/mL for BK, all within the first 6 months post-transplantation. Other secondary measures encompassed renal allograft function at the 6-month post-transplant mark, admission for transplant LOS and the total number of readmissions post-transplant during the initial 6 months post-transplant.

### Data extraction and study assessments

The following information was extracted from electronic patient records: age, gender, etiology of end-stage renal disease (ESRD), dialysis pre-transplant, donor type, age and gender, donor–recipient's degree of HLA mismatch (HLA MM) and donor/recipient CMV status, transplant date and number, induction type, ESW status, presence of delayed or slowed graft function, relevant medical history [specifically smoking status, hypertension, congestive heart failure (CHF), ischemic heart disease (IHD) , pre-transplant diabetes and pre-transplant immunosuppressive therapy], perioperative complications and the occurrence of early ACR.

The following clinical and biochemical parameters were retrieved in an automated fashion from MDClone: admission for transplant LOS, number of admissions post the transplant admission until 6 months post-transplant, average 1–6 months post-transplant systolic and diastolic blood pressures, weight and body mass index (BMI), average Scr 25–40 days post-transplant (Scr 1 m), average Scr 2.5–3.5 months post-transplant (Scr 3 m) and average Scr 5–7 months post-transplant (Scr 6 m). Average tacrolimus trough blood levels were retrieved 0–3, 0–7 and 0–30 days post-transplant and 1–6 months post-transplant. Additional data retrieved between 3–6 months post-transplant: average hemoglobin (Hb), average absolute lymphocyte count, average absolute neutrophil count, average platelets (PLT) count, average glucose and HbA1C, average uric acid, average albumin and globulin, average C-reactive protein (CRP) and average protein/creatinine ratio. Infections data obtained included: CMV and BK viremia 1–6 months post-transplant, urine and blood culture results 0–1 and 1–6 months post-transplant. Use of the following medications between 1–6 months post-transplant was automatically obtained from MDClone: MPA, mTOR inhibitor, beta blockers (BB), calcium channel blockers (CCB) and renin–angiotensin–aldosterone system (RAAS) inhibitors.

### Statistical analysis

All demographic, clinical and biochemical covariates of interest were systematically tabulated and compared. Categorical variables were compared using the Chi-squared test, with Fisher's exact test employed in cases of small cell counts. Continuous variables underwent preliminary normality testing using the Shapiro–Wilk test, alongside assessments for equality of variances. Subsequently, normally distributed variables were compared using t-tests or analysis of variance, while non-normally distributed variables were subjected to non-parametric tests.

For the primary outcome of post-transplant ACR, a logistic multivariable model was employed. Initially, univariate models were evaluated, with variables displaying significance (*P* < .05) or deemed clinically relevant progressing to multivariate modeling. Statistical analyses were performed using the SPSS software package.

## RESULTS

### Characteristics of the cohort of non-sensitized KTRs who had a first live-donor kidney transplant and received basiliximab for induction

For the 113 KTRs (Table 1A), mean transplant age was 53 ± 14.5 years, 84 (74.3%) were males and 81 (71.1%) were on renal replacement therapy before the transplant. Ninety (79.6%) had an LURD transplant while 23 (20.4%) had a living related donor (LRD) transplant. Past medical histories of hypertension, diabetes, IHD and CHF were present in 102 (90.3%), 35 (31%), 25 (22.1%) and 6 (5.3%) KTRs, respectively. Mean donor age was 41.2 ± 9.9 years and 75 (67.6%) of the donors were males. Donor/recipient CMV status was positive/positive in 86 (76.1%) and positive/negative in 12 (10.6%) KTRs. Mean admission for transplant LOS was 9.2 ± 3.5 days. Early ACR occurred in 17 (15%) of the cohort with 9 (52.9%) of the ACR occurred within the first post-transplant week. Other clinical characteristics including ESRD etiology, time on pre-transplant dialysis, rate of pre-transplant immunosuppressive therapy, perioperative complications, rate of delayed or slow graft function and of ESW, and number of readmissions post-transplant up to 6 months post-transplant are detailed in Table 1A. For all other biochemical and clinical characteristics including vital signs, Scr and estimated glomerular filtration rate (eGFR) up to 6 months post-transplant and other laboratory results, rate of infections including presence of CMV and BK viremia, UTI and bacteremia up to 6 months post-transplant and medications use, see Table 1B.

**Table 1A:** Demographic and clinical characteristics of KTRs who received basiliximab for induction stratified by degree of HLA mismatch.

Variable	Total cohort (*n* = 113)	High HLA match group 0–4 MM (*n* = 61)	Low HLA match group 5–6 MM (*n* = 52)	*P*-value
RTR characteristics				
Transplant age, years (mean ± SD)	53 ± 14.5	53.4 ± 15.9	52.5 ± 12.8	.742
Male sex, *n* (%)	84 (74.3)	45 (73.8)	39 (75.0)	.881
ESRD etiology, *n* (%)				
DN	17 (15.0)	10 (16.4)	7 (13.5)	.703
GN	29 (25.7)	16 (26.2)	13 (25.0)	
Nephrosclerosis	14 (12.4)	7 (11.5)	7 (13.5)	
PCKD	14 (12.4)	6 (9.8)	8 (15.4)	
Other	21 (18.6)	14 (23.0)	7 (13.5)	
Unknown	18 (15.9)	8 (13.1)	10 (19.2)	
Pre-transplant dialysis	81 (71.7)	44 (72.1)	37 (71.2)	.909
Time on dialysis (years) [median (IQR)]	1.2 (0.5–2.5)	1.1 (0.4–2.4)	1.3 (1.3–2.7)	.233
Smoking status				
Current smokers	8 (7.1)	3 (4.9)	5 (9.6)	.548
Past smokers	34 (30.1)	20 (32.8)	14 (26.9)	
No	71 (62.8)	38 (62.3)	33 (63.5)	
Medical history, *n* (%)				
HTN	102 (90.3)	55 (90.2)	47 (90.4)	.969
Pre-transplant diabetes	35 (31.0)	19 (31.1)	16 (30.8)	.965
IHD	25 (22.1)	12 (19.7)	13 (25.0)	.496
CHF	6 (5.3)	2 (3.3)	4 (7.7)	.411
Pre-transplant immunosuppressive therapy	21 (18.6)	12 (19.7)	9 (17.3)	.747
Donor characteristics				
Type, *n* (%)				
LURD	90 (79.6)	38 (62.3)	52 (100)	<.001**
LRD	23 (20.40)	23 (37.7)		
Donor age, years (mean ± SD)	41.2 ± 9.9	39.9 ± 9.3	42.9 ± 10.5	.113
Male sex, *n* (%)	75 (67.6)	36 (60.0)	39 (76.5)	**.065**
CMV D/R, *n* (%)				
Pos/Pos	86 (76.1)	46 (75.4)	40 (76.9)	.634
Pos/Neg	12 (10.6)	5 (8.2)	7 (13.5)	
Neg/Pos	12 (10.6)	8 (13.1)	4 (7.7)	
Neg/Neg	3 (2.7)	2 (3.3)	1 (1.9)	
Peritransplant data				
Admission for transplant LOS (mean ± SD)	9.2 ± 3.5	9.4 ± 4.5	9.0 ± 1.8	.464
Perioperative complications, *n* (%)				
Infectious	9 (8)	4 (6.5)	5 (9.6)	.923
Cardiovascular	6 (5.3)	3 (4.9)	3 (5.8)	
Surgical	7 (6.2)	5 (8.2)	2 (3.8)	
Obstructive uropathy	3 (2.7)	2 (3.3)	1 (1.9)	
Other	3 (2.7)	1 (1.6)	2 (3.8)	
Delayed graft function, *n* (%)	3 (2.7)	2 (3.3)	1 (1.9)	1
Slow graft function, *n* (%)	7 (6.2)	3 (4.9)	4 (7.7)	.701
Early ACR, *n* (%)	17 (15.0)	5 (8.2)	12 (23.1)	.027*
Days from transplant to ACR (*n* = 17), *n* (%)				
2–7	9 (52.9)	2 (40.0)	7 (59.0)	.677
≥8	8 (47.1)	3 (60.0)	5 (41.0 )	
ESW, *n* (%)	55 (48.7)	36 (59.0)	19 (36.5)	.017*
Number of admissions (up to 6 months post-transplant) [median (IQR)]	0 (0–1)	0 (0–1.5)	1 (0–1)	.998

*P < .05; **P < .01.

DN, diabetic nephropathy; GN, glomerulonephritis; HTN, hypertension; IQR, interquartile range; PCKD, polycystic kidney disease; RTR, renal transplant recipients; SD, standard deviation/R, donor/recipient; Pos, positive; Neg, negative.

**Table 1B:** Biochemical and clinical characteristics of KTRs who received basiliximab for induction stratified by degree of HLA mismatch.

Variable	Total cohort (*n* = 113)	High HLA match group 0–4 MM (*n* = 61)	Low HLA match group 5–6 MM (*n* = 52)	*P*-value
Vital signs and other clinical parameters, 1–6m, average (mean ± SD)				
SBP (mmHg)	130.9 ± 14.8	130.1 ± 13.8	131.8 ± 16.1	.543
DBP (mmHg)	76.6 ± 7.0	76.5 ± 6.5	76.6 ± 7.5	.953
Weight (kg)	77.4 ± 15.2	76.7 ± 13.6	78.0 ± 16.8	.66
BMI (kg/m^2^)	26.6 ± 4.3	26.8 ± 3.3	26.4 ± 5.1	.718
Serum creatinin (mg/dL) and eGFR (CKD-EPI) post-transplant^a^ (mean ± SD)				
Scr 1 m, average	1.4 ± 0.5	1.3 ± 0.4	1.4 ± 0.5	.681
eGFR 1 m, average	59.4 ± 17.6	59.1 ± 17.3	59.7 ± 18.0	.858
Scr 3 m, average	1.4 ± 0.4	1.3 ± 3.5	1.4 ± 0.5	.53
eGFR 3 m, average	60.4 ± 17.7	60.0 ± 16.0	60.8 ± 19.5	.81
Scr 6 m, average	1.3 ± 0.4	1.3 ± 0.4	1.4 ± 0.5	.622
eGFR 6 m, average	62.7 ± 17.2	62.8 ± 16.0	62.6 ± 18.6	.951
Other laboratory results				
Tacrolimus level 0–7 days, max (μg/L) (mean ± SD)	14.5 ± 5.4	14.3 ± 5.5	14.7 ± 5.4	.739
Tacrolimus level 0–7 days, min (μg/L) (mean ± SD)	6.2 ± 2.2	6.4 ± 2.6	6.0 ± 1.6	.401
Tacrolimus level 0–7 days, average (μg/L) (mean ± SD)	9.6 ± 2.7	9.5 ± 2.9	9.6 ± 2.5	.852
Tacrolimus level 0–30 days, average (μg/L) (mean ± SD)	10.8 ± 1.7	10.9 ± 1.9	10.6 ± 1.3	.286
Tacrolimus level 1–6 m, average (μg/L) (mean ± SD)	8.9 ± 1.1	8.7 ± 1.2	9.2 ± 1.0	.014^*^
Hb 3–6 m, average (g/dL) (mean ± SD)	13.0 ± 1.6	13.1 ± 1.5	12.9 ± 1.7	.649
Lympho 3–6 m, average (K/μL) (mean ± SD)	1.5 ± 0.5	1.4 ± 0.5	1.5 ± 0.6	.488
Neut 3–6 m, average (K/μL) (mean ± SD)	4.2 ± 1.4	4.2 ± 1.1	4.3 ± 1.6	.634
PLT 3–6 m, average (K/μL) (mean ± SD)	193.2 ± 58.9	190.3 ± 59.1	196.3 ± 59.1	.606
Glucose 3–6 m, average (mg/dL) (mean ± SD)	121.0 ± 32.6	118.7 ± 32.6	123.4 ± 32.7	.463
HbA1C 3–6 m, average (g/dL) (mean ± SD)	5.9 ± 1.1	5.8 ± 0.9	6.1 ± 1.1	.28
Uric acid 3–6 m, average (mg/dL) (mean ± SD)	6.0 ± 1.4	6.0 ± 1.1	6.0 ± 1.6	.828
Albumin 3–6 m, average (g/dL) (mean ± SD)	4.1 ± 0.3	4.1 ± 0.3	4.1 ± 0.3	.479
Globulin 3–6 m, average (g/dL) (mean ± SD)	2.6 ± 0.3	2.6 ± 0.3	2.5 ± 0.3	.193
CRP 3–6 m, average [median (IQR)]	4.5 (2.2–11.2)	4.7 (2.4–9.6)	4.6 (2.1–11.9)	.874
Urine protein/creatinine 3–6 m, average [median (IQR)]	0.2 (0.1–0.3)	0.2 (0.1–0.3)	0.2 (0.1–0.3)	.332
Infections, *n* (%)				
CMV positive (>1000 copies/mL) 1–6 m	1 (0.9)	1 (1.6)	0	1
BK positive (>500 copies/ml) 1–6 m	16 (14.2)	8 (13.1)	8 (15.4)	.73
Positive urine culture 0–1 m	14 (12.4)	8 (13.1)	6 (11.5)	.8
Positive urine culture 1–6 m	18 (15.9)	7 (11.5)	11 (21.2)	.161
Positive blood culture 0–1 m	1 (0.9)	1 (1.6)	0	.345
Positive blood culture 1–6 m	1(0.9)	1 (1.6)	0	1
Medications, *n* (%)				
MPA 1–6 m	99 (87.6)	51 (83.3)	48 (92.3)	.162
mTOR inhibitor 1–6 m	2 (1.8)	0	2 (3.8)	.21
BB 1–6 m	68 (60.2)	35 (57.4)	33 (63.5)	.51
CCB 1–6 m	56 (49.6)	29 (47.5)	27 (51.9)	.642
RAAS inhibition 1–6 m	19 (16.8)	7 (11.5)	12 (23.1)	.1

a One-month average, values measured 25–40 days post-transplant, 3-month average, values measured 2.5–3.5 months post-transplant; 6-month average, values measured 5–7 months post-transplant.

eGFR was calculated according to the following CKD-EPI formula: eGFR = 141 * min (Scr/k, 1)α * max(Scr/k, 1) – 1.209 * 0.993Age * 1.018 * 1.159 (if Black), where Scr, standardized serum creatinine; k = 0.7 if female, 0.9 if male; α = –0.329 if female, –0.411 if male; min = the minimum of Scr/k of 1; max = the maximum of Scr/k or 1.

^*^ *P* < .05.

m, month; BB, beta blockers; CKD-EPI, Chronic Kidney Disease Epidemiology Collaboration; DBP, diastolic blood pressure; IQR, interquartile range; Lympho, lymphocytes; Neut, neutrophils; PLT, platelets; SBP, systolic blood pressure; SD, standard deviation.

### Univariate comparison of non-sensitized KTRs who had a first live-donor kidney transplant and received basiliximab for induction with high HLA match (0–4 MM) vs those with low HLA match (5–6 MM)

The KTRs cohort was divided into two groups, 61 (54%) with high HLA match (0–4 MM) and 52 (46%) with low HLA match (5–6 MM). In the high HLA match group 23 (37.7%) had an LRD transplant and 38 (62.3%) had an LURD transplant, while in the low HLA match group all had an LURD transplant (*P* < .001). A higher rate of donor males was present in the low compared with the high HLA match group [39 (76.5%) and 36 (60%), respectively, *P* = .065]. Rate of early ACR was significantly higher in the high compared with the low HLA match group [12 (23.1%) and 5 (8.2%), respectively, *P* = .027]. ESW was more prevalent in the low as opposed to the high HLA match group (59% vs 36.5%, *P* = .017). All other comparisons of characteristics are shown in Table 1A.

Mean tacrolimus trough blood level at 1–6 months post-transplant was significantly higher in the low compared with the high HLA match group (9.2 ± 1.0 vs 8.7 ± 1.2 μg/L, *P* = .014), while mean tacrolimus trough blood levels were not significantly different between the groups at 0–7 days and 0–30 days post-transplant. For all other variables which were not significantly different between the two groups including CMV and BK viremia from 1–6 months post-transplant, UTI and bacteremia rate from 0–1 months and from 1–6 months post-transplant, see Table 1B.

### Characteristics of the cohort of a non-sensitized KTRs who had a first live-donor kidney transplant with low HLA match (5–6 MM)

For the 96 KTRs (Table 2A), mean transplant age was 52.7 ± 12.5 years, 69 (71.9%) were males and 74 (77.1%) were on renal replacement therapy before the transplant. Past medical histories of hypertension, diabetes, IHD and CHF were present in 87 (90.6%), 29 (30.2%), 24 (25.0%) and 5 (5.2%) KTRs, respectively. Mean donor age was 42.9 ± 9.5 years and 67 (70.5%) of the donors were males. Donor/recipient CMV status was positive/positive in 72 (75.0%) and positive/negative in 15 (15.6%) KTRs. Mean admission for transplant LOS was 9.3 ± 2.8 days. Early ACR occurred in 16 (16.7%) of the cohort with 9 (56.3%) of the ACR occurring within the first post-transplant week. Other clinical characteristics are detailed in Table 2A. For all other biochemical and clinical characteristics including vital signs, Scr and eGFR up to 6 months post-transplant and other laboratory results, rate of infections including presence of CMV and BK viremia, UTI and bacteremia up to 6 months post-transplant and medications use, see Table 2B.

**Table 2A:** Demographic and clinical characteristics of low HLA match (5–6 MM) KTRs who received basiliximab versus combined ATG–basiliximab for induction.

Variable	Total cohort (*n* = 96)	Basiliximab (*n* = 52)	ATG–basilixiamb (*n* = 44)	*P-*value
RTR characteristics				
Transplant age, years (mean ± SD)	52.7 ± 12.5	52.5 ± 12.8	53.0 ± 12.2	.84
Male sex, *n* (%)	69 (71.9)	39 (75.0)	30 (68.2)	.459
ESRD etiology, *n* (%)				
DN	15 (15.6)	7 (13.5)	8 (18.2)	.267
GN	22 (22.9)	13 (25.0)	9 (20.5)	
Nephrosclerosis	8 (8.3)	7 (13.5)	1 (2.3)	
PCKD	18 (18.8)	8 (15.4)	10 (22.7)	
Other	17 (17.7)	7 (13.5)	10 (22.7)	
Unknown	16 (16.7)	10 (19.2)	6 (13.6)	
Pre-transplant dialysis, *n* (%)	74 (77.1)	37 (71.2)	55 (84.1)	.133
Time on dialysis (years) [median (IQR)]	1.5 (0.6–3.1)	1.3 (1.3–2.7)	1.8 (0.7–3.3)	.675
Smoking status, *n* (%)				
Current smokers	10 (10.4)	5 (9.6)	5 (11.4)	.949
Past smokers	25 (26.0)	14 (26.9)	11 (25.0)	
No	61 (63.5)	33 (63.5)	28 (63.6)	
Medical history, *n* (%)				
Hypertension	87 (90.6)	47 (90.4)	40 (90.9)	1
Pre-transplant diabetes	29 (30.2)	16 (30.8)	13 (29.5)	.896
IHD	24 (25.0)	13 (25.0)	11 (25.0)	1
CHF	5 (5.2)	4 (7.7)	1 (2.3)	.371
Pre-transplant immunosuppressive therapy	14 (14.6)	9 (17.3)	5 (11.4)	.411
Donor characteristics				
Donation age, years (mean ± SD)	42.9 ± 9.5	42.9 ± 10.5	42.9 ± 8.3	.979
Male sex, *n* (%)	67 (70.5)	39 (76.5)	28 (63.6)	.171
CMV D/R, *n* (%)				
Pos/Pos	72 (75.0)	40 (76.9)	32 (72.7)	.73
Pos/Neg	15 (15.6)	7 (13.5)	8 (18.2)	
Neg/Pos	8 (8.3)	4 (7.7)	4 (9.1)	
Neg/Neg	1 (1.0)	1 (1.9)	0	
Peritransplant data				
Admission for transplant LOS (mean ± SD)	9.3 ± 2.8	9.0 ± 1.8	9.6 ± 3.6	.278
Perioperative complications, *n* (%)				
Infectious	11 (11.45)	5 (9.6)	6 (13.6)	.935
Cardiovascular	6 (6.25)	3 (5.8)	3 (6.8)	
Surgical	5 (5.2)	2 (3.8)	3 (6.8)	
Obstructive uropathy	1 (1.04)	1 (1.9)	0	
Other	4 (4.2)	2 (3.8)	2 (4.5)	
Delayed graft function, *n* (%)	5 (5.3)	1 (1.9)	4 (9.1)	.176
Slow graft function, *n* (%)	12 (12.5)	4 (7.7)	8 (18.2)	.122
Early ACR, *n* (%)	16 (16.7)	12 (23.1)	4 (9.1)	.067
Days from transplant to ACR (*n* = 17), *n* (%)				
2–7	9 (56.3)	7 (59.0)	2 (50.0)	.464
≥8	7 (43.7)	5 (41.0 )	2 (50.0)	
ESW	44 (45.8)	19 (36.5)	25 (56.8)	.047^*^
Number of admissions (up to 6 months post-transplant)	0 (0–1)	1 (0–1)	0 (0–1)	.218

* *P* < .05.

DN, diabetic nephropathy; GN, glomerulonephritis; IQR, interquartile range; PCKD, polycystic kidney disease; RTR, renal transplant recipients; SD, standard deviation; D, donor; R, recipient; Pos, positive; Neg, negative.

**Table 2B:** Biochemical and clinical characteristics of low HLA match (5–6 MM) KTRs who received basiliximab versus combined ATG–basiliximab for induction.

Variable	Total cohort (*n* = 96)	Basiliximab (*n* = 52)	ATG–basilixiamb (*n* = 44)	*P*-value
Vital signs and other clinical parameters, 1–6 m average (mean ± SD)				
SBP (mmHg)	132.4 ± 14.6	131.8 ± 16.1	133.1 ± 12.6	.673
DBP (mmHg)	78.0 ± 7.6	76.6 ± 7.5	79.8 ± 7.5	.048
Weight (kg)	78.2 ± 15.7	78.0 ± 16.8	78.4 ± 14.5	.913
BMI (kg/m^2^)	26.4 ± 4.6	26.4 ± 5.1	26.4 ± 3.8	.992
Serum creatinine (mg/dL) and eGFR (CKD-EPI) post-transplant (mean ± SD)				
Scr 1 m, average	1.4 ± 0.5	1.4 ± 0.5	1.3 ± 0.3	.544
eGFR 1 m, average	59.6 ± 17.3	59.7 ± 18.0	59.6 ± 16.6	.973
Scr 3 m, average	1.3 ± 0.4	1.4 ± 0.5	1.3 ± 0.3	.127
eGFR 3 m, average	61.4 ± 17.6	60.8 ± 19.5	62.1 ± 14.7	.73
Scr 6 m, average	1.3 ± 0.3	1.4 ± 0.5	1.2 ± 0.3	.153
eGFR 6 m, average	63.6 ± 17.5	62.6 ± 18.6	64.9 ± 16.2	.508
Other laboratory results				
Tacrolimus level 0–3 days, max (μg/L) (mean ± SD)	11.7 ± 5.7	13.0 ± 6.2	9.9 ± 4.5	.02*
Tacrolimus level 0–3 days, min (μg/L) (mean ± SD)	8.1 ± 4.0	8.9 ± 4.5	7.0 ± 2.9	.185
Tacrolimus level 0–3 days, average (μg/L) (mean ± SD)	9.8 ± 4.5	10.9 ± 5.0	8.4 ± 3.5	.006**
Tacrolimus level 0–7 days, max (μg/L) (mean ± SD)	13.5 ± 4.9	14.7 ± 5.4	11.8 ± 3.8	.005**
Tacrolimus level 0–7 days, min (μg/L) (mean ± SD)	5.8 ± 1.7	6.0 ± 1.6	5.5 ± 1.9	.348
Tacrolimus level 0–7 days, average (μg/L) (mean ± SD)	9.0 ± 2.4	9.6 ± 2.5	8.2 ± 2.1	.006**
Tacrolimus level 0–30 days, average (μg/L) (mean ± SD)	10.2 ± 1.5	10.6 ± 1.3	9.8 ± 1.5	.015**
Tacrolimus level 1–6 m, average (μg/L) (mean ± SD)	9.3 ± 1.6	9.2 ± 1.0	9.5 ± 2.1	.34
Hb 3–6m, average (g/dL) (mean ± SD)	12.9 ± 1.6	12.9 ± 1.7	12.8 ± 1.5	.71
Lympho 3–6m, average (K/μL) (mean ± SD)	1.4 ± 0.5	1.5 ± 0.6	1.3 ± 0.4	.07
Neut 3–6 m, average (K/μL) (mean ± SD)	4.3 ± 1.7	4.3 ± 1.6	4.2 ± 1.8	.754
PLT 3–6 m, average (K/μL) (mean ± SD)	201.8 ± 58.0	196.3 ± 59.1	210.2 ± 56.1	.277
Glucose 3–6 m, average (mg/dL) (mean ± SD)	118.4 ± 29.4	123.4 ± 32.7	110.8 ± 21.8	.053
HbA1C 3–6 m, average (g/dL) (mean ± SD)	5.9 ± 1.2	6.1 ± 1.1	5.7 ± 1.1	.19
Uric acid 3–6 m, average (mg/dL) (mean ± SD)	6.1 ± 1.5	6.0 ± 1.6	6.3 ± 1.3	.283
Albumin 3–6 m, average (g/dL) (mean ± SD)	4.2 ± 0.3	4.1 ± 0.3	4.2 ± 0.2	.752
Globulin 3–6 m, average (g/dL) (mean ± SD)	2.5 ± 0.3	2.5 ± 0.3	2.5 ± 0.3	.682
CRP 3–6m, average (mg/L) [median (IQR)]	4.3 (1.9–12.0)	4.6 (2.1–11.9)	4.2 (1.6–12.3)	.301
Urine protein/creatinine 3–6 m, average [median (IQR)]	0.2 (0.1–0.3)	0.2 (0.1–0.3)	0.1 (0.1–0.3)	.659
Infections, *n* (%)				
CMV positive (>1000 copies/mL) 1–6 m	2 (2.1)	0	2 (4.5)	.207
BK positive (>500 copies/mL) 1–6 m	13 (13.5)	8 (15.4)	5 (11.4)	.566
Positive urine culture 0–1 m	12 (12.5)	6 (11.5)	6 (13.6)	.757
Positive urine culture 1–6 m	17 (17.7)	11 (21.2)	6 (13.6)	.336
Positive blood culture 0–1 m	2 (2.1)	0	2 (4.5)	.207
Positive blood culture 1–6 m	0	0	0	
Medications, *n* (%)				
MPA 1–6 m	90 (93.8)	51 (98.1)	39 (88.6)	.09
mTOR inhibitor 1–6 m	3 (3.1)	2 (3.8)	1 (2.3)	1
BB 1–6 m	58 (60.4)	33 (63.5)	25 (56.8)	.507
CCB 1–6 m	47 (49.0)	27 (51.9)	20 (45.5)	.528
RAAS inhibition 1–6 m	15 (15.6)	12 (23.1)	3 (6.8)	.029*

a One-month average, values measured 25–40 days post-transplant, 3-month average, values measured 2.5–3.5 months post-transplant; 6-month average, values measured 5–7 months post-transplant.

eGFR was calculated according to the following CKD-EPI formula: eGFR = 141 * min (Scr/k, 1)α * max(Scr/k, 1) – 1.209 * 0.993Age * 1.018 * 1.159 (if Black), where Scr, standardized serum creatinine; k = 0.7 if female, 0.9 if male; α = –0.329 if female, –0.411 if male; min = the minimum of Scr/k of 1; max = the maximum of Scr/k or 1.

*P < .05; ^**^P < .01.

m, month; BB, beta blockers; BMI, body mass index; CKD-EPI, Chronic Kidney Disease Epidemiology Collaboration; DBP, diastolic blood pressure; IQR, interquartile range; Lympho, lymphocytes; MPA, mycophenolic acid; mTOR, mammalian target of rapamycin; Neut, neutrophils; PLT, platelets; SBP, systolic blood pressure; SD, standard deviation.

### Univariate comparison of non-sensitized KTRs who had a first live-donor kidney transplant with low HLA match (5–6 MM) who received basiliximab vs combined ATG–basiliximab for induction

**Figure 2:**
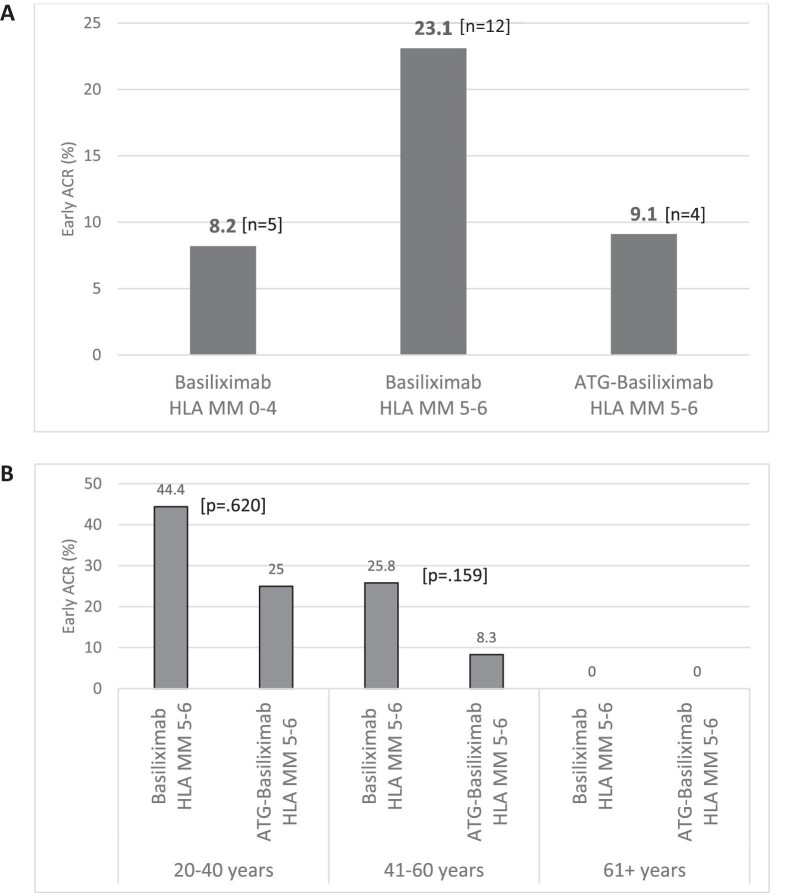
(**A**) Early ACR rates in non-sensitized KTRs with first kidney transplants, contrasting high HLA match KTRs with basiliximab and low HLA matches KTRs with basiliximab and ATG–basiliximab induction. (**B**) Early ACR rates in low HLA match KTRs receiving basiliximab alone vs ATG–basiliximab induction across age categories.

The KTRs cohort was divided into two groups, 52 (54.2%) who received basiliximab and 44 (45.8%) who were treated with combined ATG–basiliximab for induction. Early ACR rate dramatically reduced upon transition to ATG–basiliximab regimen from 12 (23.1%) to 4 (9.1%), *P* = .067 (Fig. 2A). ESW was more prevalent in the combined ATG–basiliximab as opposed to the basiliximab group (56.8% vs 36.5%, *P* = .047). All other comparisons of characteristics are shown in Table 2A.

Mean and maximum tacrolimus trough blood levels at 0–3 and 0–7 days post-transplant and mean tacrolimus trough blood level at 0–30 days post-transplant were significantly higher in the basiliximab versus combined ATG–basiliximab group. Use of RAAS inhibition medications was more prevalent in the basiliximab as opposed to the ATG–basiliximab group. For all other variables that were not significantly different between the two groups including CMV and BK viremia from 1–6 months post-transplant, UTI and bacteremia rate from 0–1 months and from 1–6 months post-transplant, see Table 2B.

### Logistic regression analysis for early post-transplant ACR in KTRs with low HLA match (5–6 MM)

In a multivariable logistic regression analysis, the odds for early ACR were 8% lower for every 1-year increase in age [odds ratio 0.92, 95% confidence interval (CI) 0.87–0.97, *P* = .002]. Induction therapy was not found to be an independent predictor for early ACR (Table 3A).

**Table 3A:** Multivariate logistic regression analysis for early post-transplant ACR in KTRs with low HLA match (5–6 MM) (*n* = 96).

Effect	Odds ratio (95% CI)	*P*-value
Age at transplant, per 1 year increase	0.92 (0.87–0.97)	.002**
Male vs female	3.24 (0.65–16.1)	.151
Combined ATG–basiliximab vs basiliximab induction	0.30 (0.08–1.11)	.07

^**^P < .01.

### Subgroup analysis of early post-transplant ACR in KTRs with low HLA match (5–6 MM) across various age groups

The low HLA group was stratified into three distinct age subgroups: 20–40 years (*n* = 17), 41–60 years (*n* = 55) and >60 years of age (*n* = 24). The rejection rate demonstrated a notable decline from 44.4% to 25% in the 20–40 years age subgroup and from 25.8% to 8.3% in the 41–60 years age subgroup. However, the *P*-values did not reach statistical significance, likely due to the limited number of recipients in each subgroup. No rejections were observed in the >60 years subgroup (Fig. 2B and Table 3B).

**Table 3B:** Univariate analysis for early ACR rate in low HLA match (5–6 MM) group divided into age subgroups.

Age subgroups	All low HLA match with early ACR, *n* (%)	Low HLA match with basiliximab early ACR, *n* (%)	Low HLA match with ATG–basilixiamb early ACR, *n* (%)	*P*-value
20–40 years (*n* = 17)	6 (35.3)	4 (44.4)	2 (25.0)	.62
41–60 years (*n* = 55)	10 (18.2)	8 (25.8)	2 (8.3)	.159
>60 years (*n* = 24)	0	0	0	

## DISCUSSION

Tailoring immunosuppressive therapy to match the immunological risk of transplant recipients poses a significant challenge. In addressing this issue, we have introduced a distinctive induction regimen that combines two well-established agents, ATG and basiliximab. This approach was specifically designed for a population of KTRs who were non-sensitized, had undergone their first kidney transplant and exhibited a low HLA match (5–6 MM).

**Figure 3:**
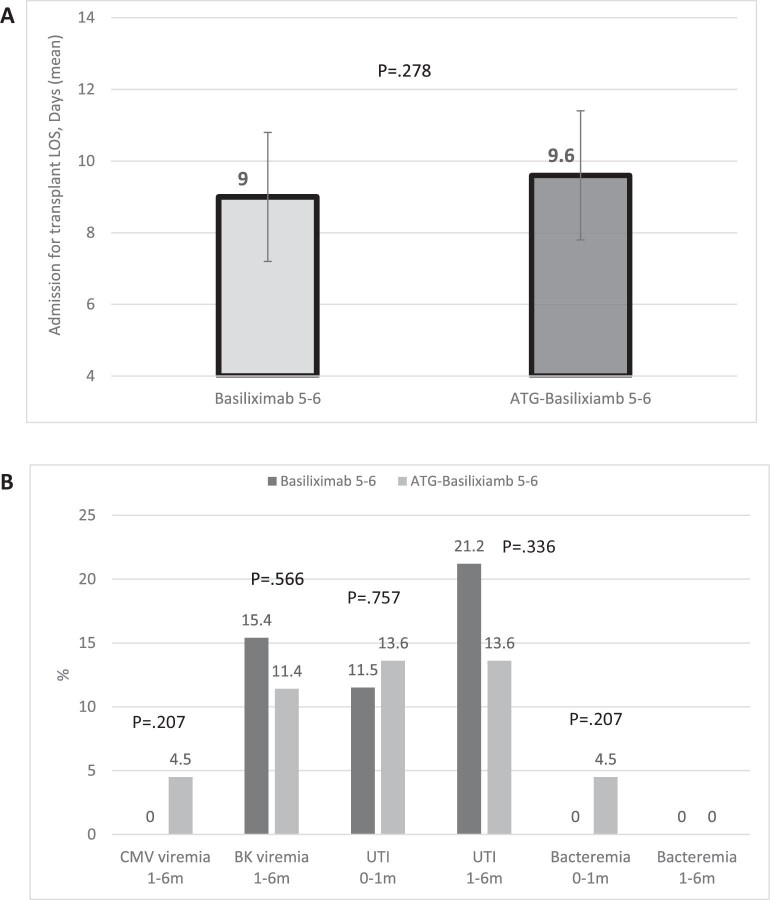
(**A**) Admission for transplant LOS in low HLA match KTRs with basiliximab vs ATG–basiliximab induction. (**B**) Infections rate up to 6 months post-transplant in low HLA match KTRs with basiliximab vs ATG–basiliximab induction.

Our findings reveal a noteworthy reduction in early ACR after changing from basiliximab to the combined ATG–basiliximab induction regimen (Fig. 2A). Although age emerged as a predominant predictor for rejection, limiting our ability to demonstrate the significance of induction therapy in a multivariable analysis, an examination of various age subgroups unveiled an even more substantial reduction in rejection rates within each group (Fig. 2B). Crucially, our study did not observe any uptick of transplant admission LOS (Fig. 3A) or perioperative complications, encompassing delayed or slow graft function, infectious complications, cardiovascular issues or any other complications. Furthermore, no increase in the incidence of post-transplant infections (Fig. 3B), or number of admissions post the transplant admission up to 6 months post-transplant was observed. Renal allograft function within the initial 6 months post-transplant demonstrated no discernible differences. Our results robustly advocate for the use of the combined ATG–basiliximab regimen for induction in this specific KTRs population, highlighting its potential safety in other KTRs.

Mitigating the incidence of acute rejection is crucial for enhancing both short- and long-term outcomes. Acute rejection is associated with worsening renal allograft function, and an augmented risk of subsequent acute and chronic rejection, thereby exacerbating long-term allograft dysfunction [16–20]. Furthermore, the potent immunosuppressive agents administered to address acute rejection are linked to an elevated short-term risk of infections [6] and a heightened long-term risk of malignancies [7, 8]. We observed an impressive reduction in rejection rate (from 23.1% to 9.1%) when switching induction therapy from basiliximab to dual ATG–basiliximab. Notably, this 9.1% rejection rate is comparable to the 8.2% rejection rate observed in the high HLA match group that received basiliximab (Fig. 2A). The diminished rejection rate was attained despite the notably lower tacrolimus 12-h trough levels observed in the combined ATG–basiliximab group during the initial 3 days, 1 week and 1 month following transplantation (Table 2B). Additionally, ESW was more frequently encountered in this group, with a prevalence of 56.8% compared with 36.5% in the basiliximab group (*P* = .047) (Table 2A). Furthermore, among the age subgroups of 20–40 and 41–60 years, the decline in rejection rate was even higher (from 44.4% to 25% and from 25.8% to 8.3%, respectively) (Table 3B).

**Figure 4:**
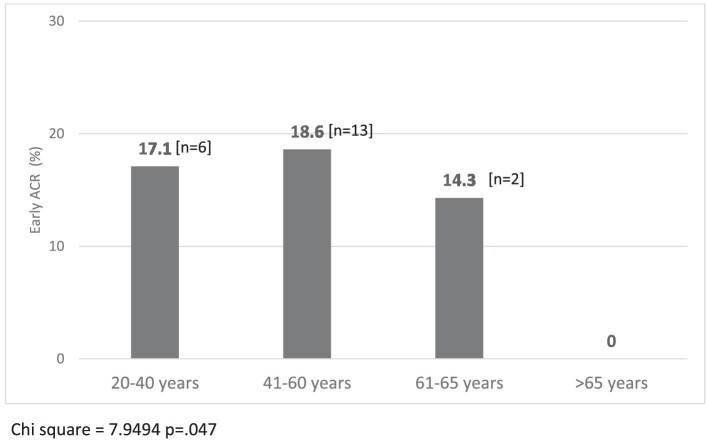
Early ACR rates in non-sensitized KTRs with first kidney transplants across age categories.

Studies exploring the association between age and rejection have revealed nuanced patterns that underscore the impact of age on graft outcomes. Younger recipients often face an increased risk of acute rejection following renal transplantation which may be attributed to a more robust immune system and increased immunogenicity, predisposing them to a more vigorous rejection response. Conversely, while older age is generally associated with a less reactive immune system, comorbidities commonly associated with aging can complicate the post-transplant course [21–23]. Our findings reveal that recipient age independently predicts rejection, with the odds of rejection decreasing by 8% for every additional year of age in a multivariate analysis (*P* = .002, Table 3A). Within our total cohort of 157 non-sensitized KTRs undergoing their first live-donor kidney transplant, the rejection rates were 17.1%, 18.6%, 14.3% and 0% for age subgroups 20–40, 41–60, 61–65 and >65 years, respectively (*P* = .047) (Fig. 4). In the subset of 96 KTRs with low HLA match (5–6 MM), rejection rates were 35.3%, 18.2% and 0% for age subgroups 20–40, 41–60 and >60 years, respectively (*P* = .01, Table 3B). Optimization of immunosuppressive approaches based on age-related factors, such as immune senescence or the heightened immune activity observed in younger recipients, should be further explored aimed at optimizing graft outcomes across diverse age groups.

Several studies have demonstrated the superior efficacy of thymoglobulin compared with interleukin-2 receptor antibodies in kidney transplant outcomes [3] revealing a lower incidence of acute rejection at 1 year post-transplant [2, 24] and a significant reduction in delayed graft function [4]. On the other hand, some studies in low immunological risk recipients have found both induction therapies to be equally effective in terms of graft and patient survival, as well as preventing acute rejection. However, ATG is associated with a higher incidence of adverse events, including CMV infection, leukopenia and thrombocytopenia [6, 25].

The optimal ATG dose remains a topic of debate, with changes observed over time. Notably, an increased ATG dose has been linked to a higher rate of complications [26]. A single high ATG dose of 9 mg/kg has been associated with significant hemodynamic and pulmonary side effects during drug infusion [4], while ATG doses lower than 5 mg/kg have been associated with a heightened risk of rejection [27]. Currently, the trend is towards shorter treatment durations (3–5 days) and lower doses (4.5–6 mg/kg) compared with older regimens which used up to 10 mg/kg for thymoglobulin [28]. In this context, a single thymoglobulin dose of 1.5 mg/kg in live-donor renal transplants has shown favorable outcomes without increasing the risk of infections or malignancies [29].

Against this backdrop, the implementation of a dual induction regimen, combining ATG and basiliximab, particularly with a single 1.5 mg/kg dose of ATG, emerges as a promising approach. This dual action targets different pathways in the immune response, potentially providing more comprehensive suppression of rejection-related immune activity, resulting in enhanced immunosuppressive efficacy, reduced rejection risk, and improved long-term graft function. Importantly, using a single 1.5 mg/kg dose of ATG in this dual induction regimen may help mitigate adverse events associated with higher doses, including infection and cytokine release syndrome, contributing to a more favorable safety profile. Additionally, from a cost-effectiveness standpoint, the dual induction regimen with a single 1.5 mg/kg ATG dose appears to strike a balance compared with other intensive induction strategies.

Our study acknowledges several limitations. While our unique induction regimen is designed for non-sensitized individuals with a low HLA match (5–6 MM) in their first kidney transplant, generalizing findings to a diverse transplant population may be limited. Uncertainties arise from the lack of consensus on the optimal ATG dose, considering variations in dosing strategies and changes over time. The study's focus on short-term outcomes within the initial 6 months post-transplant provides valuable insights, but an extended follow-up is crucial for a comprehensive evaluation. The lack of histological confirmation for some of the diagnosed rejections, retrospective design and a relatively small sample size are additional limitations. Despite these constraints, our study highlights the potential benefits of the dual low ATG dose–basiliximab induction regimen, emphasizing the need for further research and personalized induction strategies for diverse transplant cohorts.

Representing a pioneering use of the dual low ATG dose–basiliximab induction regimen, our research demonstrates its efficacy in lowering acute rejection rates without an increase in perioperative or infectious complications. In conclusion, our findings underscore the potential advantages of this induction regimen for non-sensitized KTRs undergoing their initial transplant with a l HLA match. The observed reduction in early acute rejection rates and the favorable safety profile provide encouraging evidence for the efficacy of this innovative induction approach.

## Data Availability

The data underlying this article will be shared on reasonable request to the corresponding author.
